# The Feasibility of a Fast Liver MRI Protocol for Lesion Detection of Adults at 3.0-T

**DOI:** 10.3389/fonc.2021.586343

**Published:** 2021-08-04

**Authors:** Jing Li, Chao Ma, Yukun Chen, Caixia Fu, Xinrui Wang, Bernd Kuehn, Qingsong Yang, Jianping Lu

**Affiliations:** ^1^Department of Radiology, Changhai Hospital of Shanghai, Naval Medical University, Shanghai, China; ^2^Application Developments, Siemens Shenzhen Magnetic Resonance Ltd., Shenzhen, China; ^3^Oncology Applications Predevelopment, Siemens Healthcare, Erlangen, Germany

**Keywords:** Fast, liver, MRI, workflow, auto

## Abstract

**Purpose:**

To investigate the feasibility of a fast liver magnetic resonance imaging (MRI) protocol for lesion detection in adults using 3.0-T MRI.

**Methods:**

A fast liver MRI exam protocol was proposed. The protocol included motion-resistant coronal T2-w sequence, axial T2-w fast spin echo sequence with fat suppression, axial in-op phase gradient recalled echo (GRE) T1, axial diffusion weighted imaging (DWI), and axial contrast-enhanced T1 sequences. To evaluate the diagnostic capacity of the proposed protocol, 31 consecutive patients (20 males and 11 females; mean age, 53.2 years) underwent a liver MRI exam with conventional sequences, including the proposed protocol as a subset. Images from the conventional protocol and extracted abbreviated protocol were independently read, and the diagnostic concordance rate was assessed for each patient. The concordance analysis is presented as the proportion of concordant cases between the two protocols.

**Results:**

The net measurement time of the fast liver MRI protocol without adjustment and waiting time were 4 min and 28 s. In the 31 patients included in this study, 139 suspicious findings were found from both the conventional liver MR protocol and the fast liver MRI protocol. The diagnostic concordance rate was 96.4%.

**Conclusions:**

The fast liver MRI protocol is feasible at 3.0-T, with a shorter exam time and high diagnostic concordance compared to the conventional liver MRI workflow.

## Introduction

Magnetic resonance imaging (MRI) is a well-established liver imaging modality in clinical practice, with the advantages of higher soft-tissue contrast and a lack of ionizing radiation exposure (1–4). By multi-parametric capabilities, such as T1-weighted, T2-weighted, and diffusion-weighted acquisition, MRI could offer the radiologist a more comprehensive evaluation of the liver characteristics compared to other imaging modalities, such as ultrasonography and computed tomography (CT) (5).

However, MRI has several constraints including a higher cost, longer scanning time, and a higher demand for patient cooperation (4). Due to the inherent mechanism of MRI data acquisition, respiratory triggering or breath-holding is typically needed to prevent motion artifacts during the liver exams. Moreover, due to the special hemodynamic features of some liver diseases, the contrast agent injection is essential for a successful diagnosis of liver disease. An efficient and effective imaging protocol is crucial to ensure the success of liver MRI exams. Accurate diagnosis of disease, improved patient comfort, and increased daily throughput are the expected results (1, 3). Consequently, it will help to reduce the cost of the MRI exam and ease the pressure caused by the large patient population.

With the improvement of both hardware and software, the speed of MRI and the simplicity of operation have significantly improved within the past decades. Various parallel imaging techniques, including sensitivity encoding (SENSE), array spatial sensitivity encoding technique (ASSET), generalized auto-calibrating partially parallel acquisitions (GRAPPA), and controlled aliasing in parallel imaging results in higher acceleration (CAIPIRINHA) were proposed (4, 6). These techniques significantly accelerated the use of MRI in clinical practice. For liver MRI examinations, high-quality and efficiency are continuing goals especially for clinical practice. In the current study, we propose a fast liver MRI protocol that is applicable for adult patients.

## Patients and Methods

### Patients

This study was approved by the institutional review board, and written informed consent was obtained from each enrolled patient. Thirty-one patients (20 males and 11 females; mean age, 53.2 years) were consecutively enrolled between August 2017 and September 2017. The clinical characteristics of all the 31 patients are summarized and presented in **Table 1**.

**Table 1:** Clinical characteristics of the patients.

Admitting diagnosis/medical history	No. of cases (%)
Intestinal neoplasms	13 (41.9)
Hepatic mass	2 (6.5)
Hemangioma	1 (3.2)
HCC	9 (29.0)
Carcinoma of gallbladder	1 (3.2)
Pelvic mass	1 (3.2)
Gastric carcinoma	3 (9.7)
Pancreatic cancer	1 (3.2)
Sub total	31 (100)
Age (year)	
Male*	53.35 ± 11.77
Female*	52.64 ± 8.54

*Data are means ± standard deviations.

### MRI Protocol

MRI exams were performed on a 3.0-T scanner (MAGNETOM Skyra, Siemens Healthcare, Erlangen, Germany) with a phased-array 18-channel body coil. For MRI examinations of the 31 patients, conventional liver MRI protocol was used in the study, and the detailed acquisition parameters of MRI sequences are summarized in **Table 2**. For each patient, 0.2 mmol/kg of gadopentetate dimeglumine injection was administered using an automatic injector at a flow rate of 2.0 ml/s. Following contrast injection, 15 ml of saline was injected at a flow rate of 1.0 ml/s.

**Table 2:** MRI parameters of the fast liver MRI scan and conventional liver MRI scan.

Sequences	Plane	TR (ms)	TE (ms)	Flip angle (degree)	FOV (mm^2^)	Matrix	Thickness (mm)	No. of slice	Parallel imaging Acceleration factor	Scan time
T2W HASTE*	Coronal	1000	97	160	400 × 340	256 × 240	6	15	3	15 s
T2W FS BLADE*	Axial	4000**	79	140	380 × 380	320 × 320	6	28	2	2 min, 26 s
T2W FS TSE	Axial	3000	100	160	380 × 285	320 × 288	6	28	2	1 min
DIXON VIBE*	Axial	3.97	1.26/2.49	9	400× 320	320 × 195	3	64	3	15 s
FS EPI DWI (b = 50, 1000) with 3D diagonal diffusion mode*	Axial	2200	55	90	395 × 300	128 × 96	6	20	2	38 s
Pre-contrast FS T1 VIBE	Axial	3.65	1.3	12	400 × 320	320 × 195	3	64	3	14 s
Post-contrast FS T1 VIBE*,^#^	Axial	3.65	1.3	12	400 × 320	320 × 195	3	64	3	14 s
Post-contrast FS T1 VIBE	Coronal	4.21	1.35/2.58	12	450 × 450	320 × 288	2	104	6	14 s

*All sequences in the table were used in the conventional liver scan, whereas the items marked with * were used in the abbreviated protocol. For the DWI sequence, b = 50 s/mm^2^ and 1000 s/mm^2^ were both acquired in the conventional liver scan, whereas only b = 1000 s/mm^2^ was acquired in the abbreviated protocol.

**TR is an assumed respiration period of the human adult. The actual TR varied with the actual respiration period of the patients.

^#^Post-contrast FS T1 VIBE was acquired three times.

**Figure 1:**
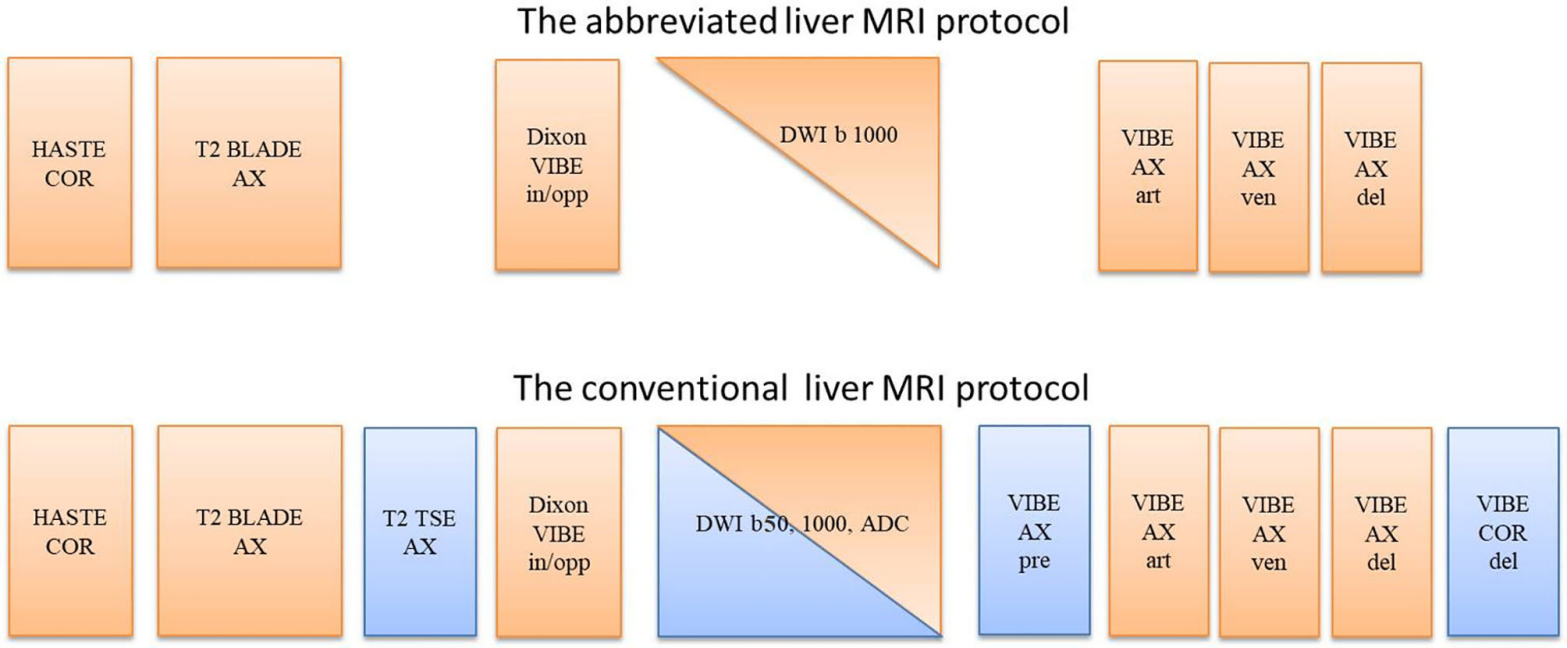
The sequences used in the abbreviated fast liver MRI protocol and the conventional liver MRI protocol. COR, coronal; AX, axial; in/opp, in-phase and opposed-phase; art, arterial phase; ven, venous phase; del, delayed phase.

The proposed fast liver MRI protocol, which was extracted from conventional protocol, included the following core sequences: localizer with three orthogonal orientations in one breath-hold; coronal T2-weighted half-Fourier single shot turbo-spin echo (HASTE) in one breath-hold; axial T2-weighted fat-saturated (FS) rotating blade-like k-space covering (BLADE) with respiratory triggering; T1-weighted volumetric interpolated breath-hold examination (VIBE) with two echoes and water-fat Dixon reconstruction in one breath-hold, acting in two roles, including the in-and-opposed phase T1-weighted, and pre-contrast T1-weighted fat-saturated scan (Dixon water image); Echo-planar-imaging (EPI) diffusion-weighted sequence with b = 1000 s/mm^2^; and three-phase (arterial, venous, and delayed phases) contrast-enhanced T1-weighted FS VIBE. **Figure 1** shows an overview of the sequences used in the abbreviated protocol and the conventional protocol, respectively.

### Data Analysis

The diagnostic concordance between the abbreviated protocol used in the fast liver workflow and the conventional protocol was evaluated using the following steps:

In step 1, the images from the whole conventional liver MRI scans were sent to the picture archiving and communication system (PACS) system and read using routine clinical procedure, i.e., a randomly assigned radiologist read the images and made a diagnostic report for each case. Another senior radiologist was randomly assigned to verify the report. In the study, liver reporting & data system (LI-RADS) assessment system (2017) was used for assigning suspicion categories of liver lesions (7). Subtracted images were not performed in our clinical workflow for liver MRI diagnosis.

In step 2, over 4 weeks after the images of the conventional liver MRI scans had been read, the images of the abbreviated protocol were extracted from the conventional liver MRI scans for the 31 patients and evaluated using the same procedure as in step 1. Thus, a second group of the reports was created.

All the radiologists in steps 1 and 2 were able to access the medical history of the patients during image reading but were blinded to the study. Additionally, radiologists in step 2 were blinded to the diagnostic report created in the step 1.

In step 3, a radiologist compared the diagnostic reports. Every suspicious finding appearing in the reports was listed side by side for both groups. The primary concordance was observed if each finding coincided with the fast liver MRI protocol and conventional liver MRI protocol regarding distribution and imaging findings. Discordance was assigned for those findings that only appeared in the report for the fast liver MRI protocol, only appeared in the conventional protocol, or were described as different imaging findings between two groups.

In step 4, for the discordant findings from step 3, a second run of the image reading was conducted by another two radiologists. Each radiologist was assigned to read the images of one group blindly, check on the presence or absence of the finding that only appeared in the report of another group, and confirm the imaging findings of those who had been assigned as different imaging findings in step 3.

In step 5, a radiologist summarized the results of steps 4 and 3 to make a final concordance report.

### Statistical Analysis

The concordance analysis was presented as the proportion of concordant cases between the two protocols for detection of the suspicious findings in the abdomen area.

## Results

**Figure 2:**
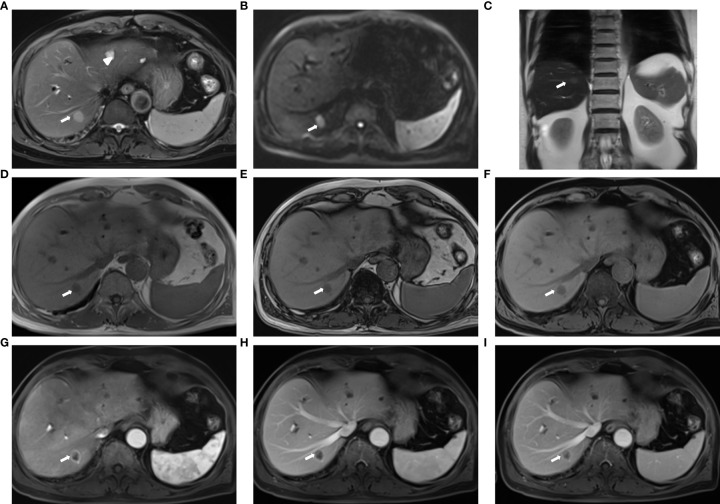
Sample images acquired with the fast liver workflow of a 74-year-old man with liver metastasis (primary: colon cancer): **(A)** T2-weighted BLADE; **(B)** DWI, b = 1000 s/mm^2^; **(C)** T2-HASTE; **(D, E)** in-phase and out-of-phase DIXON VIBE; **(F)** water images of DIXON VIBE; **(G–I)** contrast-enhanced T1WI VIBE with arterial-phase **(G)**, venous-phase **(H)**, and delayed-phase **(I)**. The metastasis (arrow) in liver segment VII appeared as slightly high signal intensity on the T2 BLADE and T2 haste image, whereas the hepatic cysts [arrowhead in **(A)**] in liver segments II and IV show high signal intensity in T2 BLADE. The metastasis is hyperintense in DWI and has low intensity in T1WI. Annular enhancement is visible in the enhanced MR imaging relative to the surrounding liver parenchyma.

All MRI images for the 31 patients had good image quality and no obvious artifacts. Sample images of the fast liver MRI protocol are shown in **Figure 2**, which demonstrates the high lesion delineation capability for liver metastasis.

### Procedure time

The net measurement time of the conventional liver MR scan and the fast MRI workflow without adjustment and waiting time was 6 min and 4 min and 28 s, respectively. In total, 13 breath-holds were used in the conventional liver scan, whereas only 7 breath-holds were required in the fast liver MRI workflow.

### Diagnostic Concordance

**Figure 3:**
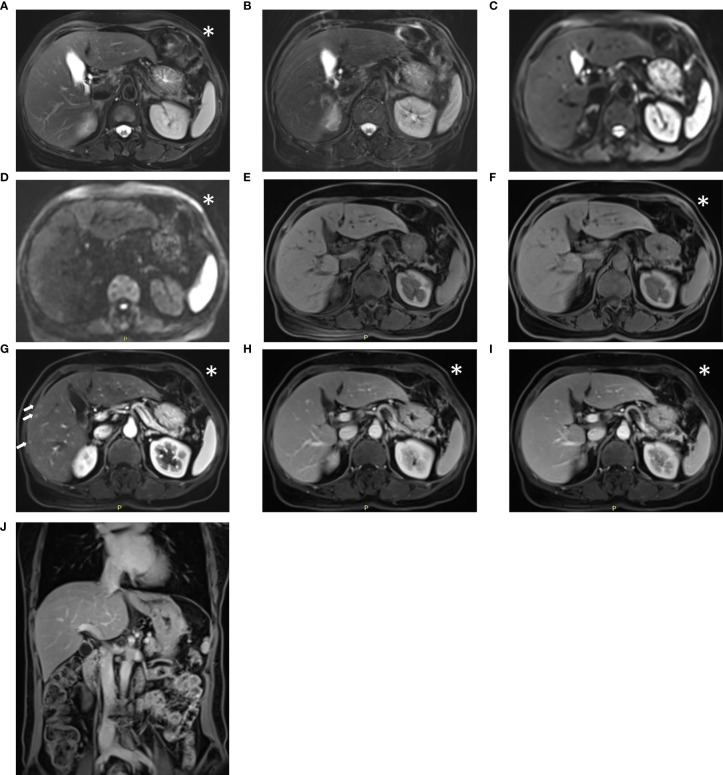
Sample images acquired with the conventional liver workflow of a 52-year-old post-surgical colon cancer patient. The lesion could not be identified on the T2-weighted BLADE image **(A)**, T2-weighted TSE image **(B)**, DW images with b = 50, 1000 s/mm^2^ **(C, D)**, pre-contrast T1-weighted Fat-Sat VIBE images **(E)**, or water images of the DIXON VIBE **(F)**. In the arterial phase T1-weighted VIBE **(G)**, multiple small hypervascular focal lesions appear in the right lobe. The suspicious lesions return to isointensity on the venous-phase **(H)** and delayed-phases **(I, J)**. The diagnostic report from the conventional protocol considered them as transient hepatic intensity difference, whereas in the report from the abbreviated protocol, these suspicious lesions were not mentioned. The abbreviated protocol acquired the image marked with an asterisk (*), whereas the conventional protocol acquired all the series. Notably, the suspicious transient hepatic intensity difference is subtle and difficult to identify.

A total of 139 suspicious findings were seen in the 31 patients. All 139 findings were reported using the conventional protocol. From the fast protocol, 134 findings were reported, with a concordance rate of 96.4%. The two reports with absences in the abbreviated fast protocol were obviously missed diagnosis after confirmation. The other three discordant cases were caused by the different imaging findings described in the pair of reports (**Figure 3**). It is not certain which report was correct from imaging because there was no gold standard at that time. **Tables 3** and **4** show more details of the suspicious findings and the results of the concordance analyses.

**Table 3:** Distribution of suspicious findings for patients.

Location of the suspicious findings	No. of data dets	Percentage
Liver	48	34.5%
Renal	23	16.5%
Gallbladder	12	8.6%
Spleen	10	7.2%
Abdominal cavity	5	3.6%
Portal vein	5	3.6%
Subcapsular area of liver	4	2.9%
Lung	4	2.9%
Stomach	3	2.2%
Pancreas	2	1.4%
Peritoneum	2	1.4%
Portahepatis	2	1.4%
Retroperitoneal space	2	1.4%
Vertebra	2	1.4%
Retroperitoneal space	2	1.4%
Bile ducts	2	1.4%
Others*	11	7.9%
Total	139	100%

*Others include those locations that have only one data set. These include adrenal, atrium, hepatic vein, lesser omentum, mesentery, paracaval, perihepatic, thoracic cavity, ureter, subdiaphragm, and vena cava.

**Table 4:** Results of concordance analysis between the two liver protocols for the detection of suspicious findings.

Suspicious findings	No. of concordant pairs	No. of discordant pairs
Cysts	32	1
Metastasis	23	2
Splenomegaly	7	0
Transient hepatic intensity difference	6	1
Tumor embolus	6	0
Cirrhosis	5	0
Cholecystolithiasis	5	0
Ascites	5	0
Hemangioma	4	0
Lymph node enlargement	3	0
hepatocellular carcinoma (HCC)	3	0
Viable residual tumor tissue of HCC after treatment	3	1
Viable residual tumor tissue of liver metastasis after treatment	2	0
Nodule	2	0
Pneumonia	2	0
Fatty liver	2	0
Chronic cholecystitis	2	0
Cholecystitis	2	0
Bile ducts dilation	2	0
Others*	18	0
**Total**	134	5
**Percentage**	96.40%	3.60%

*Others include those findings found in only one case. These include adenomyomatosis, atelectasis, cortical adenoma, esophageal-gastric varices, gallbladder enlargement, gastric antrum cancer, gastric retention, hydronephrosis, hydrothorax, hydroureterosis, infarction, pancreatic atrophy, portal, hypertension, renal atrophy, splenculus, thrombus, unviable post-treatment tumor tissue, and vascular malformation.

## Discussion

The results of the current study suggest that the proposed fast liver MR workflow is feasible in clinical practice for adults, with a shorter exam time and high diagnostic concordance compared to the conventional liver MRI workflow. The reduction of the fast liver MRI workflow measurement time was primarily attributable to the use of the abbreviated protocol. Although some discordant pairs were evident, the two reports with absences in the abbreviated protocol were obviously missed diagnosis after careful confirmation, which was not due to the omission of conventional sequences. Additionally, the involved suspicious findings were inconspicuous and difficult to identify for other three discordant cases. A possible explanation for these discordant cases may be related to the different experience or knowledge of the radiologists. In **Figure 3**, one of the discordant cases with suspicious transient hepatic intensity difference was presented. Notably, the suspicious findings that only appeared on the arterial-phase of T1-weighted image are subtle and easily missed even with the conventional liver MRI protocol. So, it also was not due to the omission of conventional sequences.

In the current study, we used a fat saturated BLADE sequence for T2-weighted imaging, instead of including both a BLADE and TSE sequence. Some studies have been reported supporting the role of the BLADE technique for reducing motion artifact and improving lesion detection for T2-weighted imaging of the liver (8–13). We also acquired opposed-phase, in-phase images with one VIBE sequence and achieved water-only images with Dixon reconstruction, which eliminated the need for a separate pre-contrast T1-weighted acquisition. For diffusion weighted imaging (DWI), only a b-value of 1000 s/mm^2^ was used in the fast liver MRI workflow. Although several previous studies reported that apparent diffusion coefficient (ADC) measurement might be a valuable diagnostic tool for the characterization of focal liver lesions (FLLs) (14, 15), other studies claimed that ADC values varied in diffuse liver diseases such as cirrhosis and in FLLs such as hemangioma, metastasis, and hepatocellular carcinoma (HCC) (16–22). In these studies, different scanners, b values, and diffusion sequences might lead to different ADC values for normal liver parenchyma and FLLs, various cutoff values in differentiating malignant and benign lesions, and overlapping ADC values for malignant and benign FLLs. Thus, ADC maps were ignored in the fast liver workflow. We applied the CAIPIRINHA technique to increase the performance acceleration technology (PAT) acceleration factor for the VIBE sequence to achieve short scan times, high spatial resolution, as well as high image quality. Several studies reported that the CAIPIRINHA technique could provide improved image quality and higher diagnostic confidence of focal liver lesions, compared to conventional parallel imaging techniques (23–26).

The high diagnostic concordance rate of the abbreviated protocol used in the fast liver MRI workflow revealed that the abbreviated protocol could provide enough diagnostic information for most of the abdominal lesions. Besides shortening the exam time, the use of the abbreviated protocol could also increase the patient’s comfort, because fewer breath-holds were needed. Moreover, as four redundant image sets were excluded (T2W TSE, ADC map, additional pre-contrast axial T1W VIBE, and delayed-phase coronal T1 VIBE), fewer images had to be read. Thus, the time spent on image reading for radiologists could be reduced, and the throughput of the diagnosis work could be improved.

Some limitations of our study should be noted. First, due to the lack of a biopsy or surgical pathology as a gold standard to assess the diagnostic efficiency, we only evaluated the diagnostic concordance between the abbreviated protocol used in the fast liver MRI workflow and the conventional MRI protocol. Second, radiologists who read the two group images in the diagnostic concordance step were randomly assigned according to daily schedule, which might have caused additional discordance due to the readers’ different experience or knowledge. Although we conducted two levels of the concordance analysis to minimize human error, unwanted discordance might have still existed. Third, our study was carried out in a single institution with a small sample size, resulting in the heterogeneity of the patient population and the absence of a separate analysis of patients with diffuse liver disease such as cirrhosis. Further study should be performed to demonstrate the efficiency of the fast protocol with larger sample size.

## Conclusions

The results show that the proposed fast liver MRI protocol is feasible in clinical practice at 3.0-T. This modality could serve to accelerate abdominal MRI in daily clinical practice, without significantly impacting diagnostic value.

## Data Availability Statement

The original contributions presented in the study are included in the article/supplementary material. Further inquiries can be directed to the corresponding authors.

## Ethics Statement

The studies involving human participants were reviewed and approved by Changhai Hospital Ethics committee. The patients/participants provided their written informed consent to participate in this study. Written informed consent was obtained from the individual(s) for the publication of any potentially identifiable images or data included in this article.

## Author Contributions

The conception and design of the study: CM, JL, and CF. Acquisition of data: JL, YC, XW, and QY. Analysis and interpretation of data: JL, CF, JPL, XW, and BK. Drafting the article: CM and JL. Final approval of the version to be submitted: JL, CM, YC, CF, BK, XW, and JPL. All authors contributed to the article and approved the submitted version.

## Funding

This work was supported by the Key junior college of national clinical of China; National Natural Science Foundation of China (81601468); Project of precision medical transformation application of SMMU (2017JZ42); Science and Technology Innovation Foundation of Shanghai (17411952200).

## Conflict of Interest

Author CF was employed by the company Siemens Shenzhen Magnetic Resonance Ltd. Author BK was employed by the company Siemens Healthcare.

The remaining authors declare that the research was conducted in the absence of any commercial or financial relationships that could be construed as a potential conflict of interest.

## Publisher’s Note

All claims expressed in this article are solely those of the authors and do not necessarily represent those of their affiliated organizations, or those of the publisher, the editors and the reviewers. Any product that may be evaluated in this article, or claim that may be made by its manufacturer, is not guaranteed or endorsed by the publisher.

## Abbreviations

MRI, magnetic resonance imaging; CT, computed tomography; SENSE, sensitivity encoding; ASSET, array spatial sensitivity encoding technique; GRAPPA, generalized autocalibrating partially parallel acquisitions; CAIPIRINHA, controlled aliasing in parallel imaging results in higher acceleration; Dot, daily optimizing throughput; HASTE, half Fourier single shot turbo-spin echo; FS, fat-saturated; BLADE, rotating blade-like k-space covering; VIBE, volumetric interpolated breath-hold examination; FLASH, fast low angle shot; DWI, diffusion weighted imaging; ADC, apparent diffusion coefficient; PACS, picture archiving and communication system; ICC, intraclass correlation coefficients; FLL, focal liver lesion; HCC, hepatocellular carcinoma; MPR, multi-planar reconstruction; PAT, performance acceleration technology.
